# Development and validation of a risk prediction model for hospital mortality in adult patients with *Staphylococcus aureus* bacteremia

**DOI:** 10.3389/fcimb.2026.1849135

**Published:** 2026-06-18

**Authors:** Hua-Zheng Guo, Zhen-Chuan Xing, Ting Ao, Ming Hu, Peng Zhen

**Affiliations:** 1 Department of Infectious Disease, Beijing Luhe Hospital, Capital Medical University, Beijing, China; 2 Department of Pulmonary and Critical Care Medicine, Beijing Luhe Hospital, Capital Medical University, Beijing, China

**Keywords:** in-hospital mortality, nomogram, prediction model, SOFA score, *Staphylococcus aureus* bacteremia

## Abstract

**Background:**

*Staphylococcus aureus* bacteremia (SAB) is a life-threatening infection with high mortality. Early identification of high-risk patients remains challenging. This study aimed to develop a prediction model for in-hospital mortality in SAB patients and compare its performance with the SOFA score.

**Methods:**

This single-center retrospective study enrolled 160 patients with SAB (2019-2024). Patients were categorized into survival (n=87) and non-survival (n=73) groups. Variable selection was performed using best subset, stepwise, and LASSO regression. A multivariable logistic regression model was developed and internally validated using bootstrap resampling. A nomogram incorporating six predictors (PaO_2_/FiO_2_ ratio, urea, albumin, RDW, MV, and platelet count) was constructed to provide individualized risk prediction. Model performance was compared with the SOFA score using ROC curves, calibration plots, and decision curve analysis.

**Results:**

The in-hospital mortality rate was 45.6%. Independent risk factors included invasive mechanical ventilation (OR 4.738), elevated RDW (OR 1.252), and elevated urea (OR 1.065), while higher platelet count (OR 0.991), albumin (OR 0.825), and PaO_2_/FiO_2_ ratio (OR 0.988) were protective (all *P* < 0.05). The model showed excellent discrimination (AUC 0.948) and calibration (Hosmer-Lemeshow *P* = 0.658). The SOFA score showed comparable discrimination (AUC 0.959) but suboptimal calibration (*P* = 0.026). No significant difference in AUC was observed (*P* = 0.565).

**Conclusions:**

Invasive mechanical ventilation, elevated RDW, and elevated urea are independent risk factors for mortality in SAB, while higher platelet count, albumin, and PaO_2_/FiO_2_ ratio are protective. The proposed nomogram provides excellent predictive performance comparable to the SOFA score and may aid early identification of high-risk patients.

## 1 Introduction

*Staphylococcus aureus* bacteremia (SAB) is a common invasive infection in clinical practice and represents a leading cause of bacteremia-related morbidity and mortality worldwide. The case fatality rate of SAB ranges from 15% to 30%, with an estimated 300,000 deaths occurring annually (Tong et al., 2025). The prognosis of SAB is influenced by multiple factors, including underlying host comorbidities, pathogen antimicrobial resistance, source control of infection, and the host’s inflammatory response and organ function status. Mortality is further increased particularly in patients with concomitant septic shock or multiple organ dysfunction. A systematic review and meta-analysis involving 536,791 patients reported that the 1-month case fatality rate for SAB was 18.1% in the period after 2011, rising to 27.0% at 3 months. Notably, patients with methicillin-resistant *Staphylococcus aureus* (MRSA) bacteremia exhibited a significantly higher mortality risk compared to those with methicillin-susceptible *Staphylococcus aureus* (MSSA) bacteremia (Bai et al., 2022). A single-center retrospective study involving 356 patients with SAB demonstrated that leukemia, pneumonia, and concomitant sepsis were independent risk factors for 30-day mortality. Specifically, pneumonia was associated with an approximately 15-fold higher risk of death, while leukemia conferred a 28-fold increased risk. Furthermore, the neutrophil-to-lymphocyte ratio, C-reactive protein, and albumin levels were also found to be closely correlated with short-term mortality (Borcak et al., 2025). Another multicenter cohort study further revealed the existence of distinct clinical subphenotypes among patients with SAB. Among these, the subphenotype of hospital-acquired catheter-related bacteremia was associated with a favorable prognosis, whereas the subphenotype of community-acquired metastatic infection exhibited a higher risk of complications and worse microbiological outcomes (Swets et al., 2024). Early identification of high-risk patients and implementation of targeted interventions are key to improving prognosis. Currently, the Sequential Organ Failure Assessment (SOFA) score is commonly used in clinical practice to assess disease severity (Singer et al., 2016). However, this scoring system was not specifically designed for SAB, and its discriminatory performance differs by infection type and patient subgroup, demonstrating inconsistency across diverse populations (Sparks et al., 2022; Liu et al., 2024). Developing a prediction model for mortality in patients with SAB and performing individualized risk assessment may facilitate early identification of patients at high risk of death and timely adjustment of treatment decisions. This study aims to analyze clinical data of patients with SAB, develop a prediction model for in-hospital mortality, and compare its predictive performance with the SOFA score, thereby providing a reference for early identification and intervention in high-risk patients.

## 2 Methods

### 2.1 Study population and objectives

**Figure 1:**
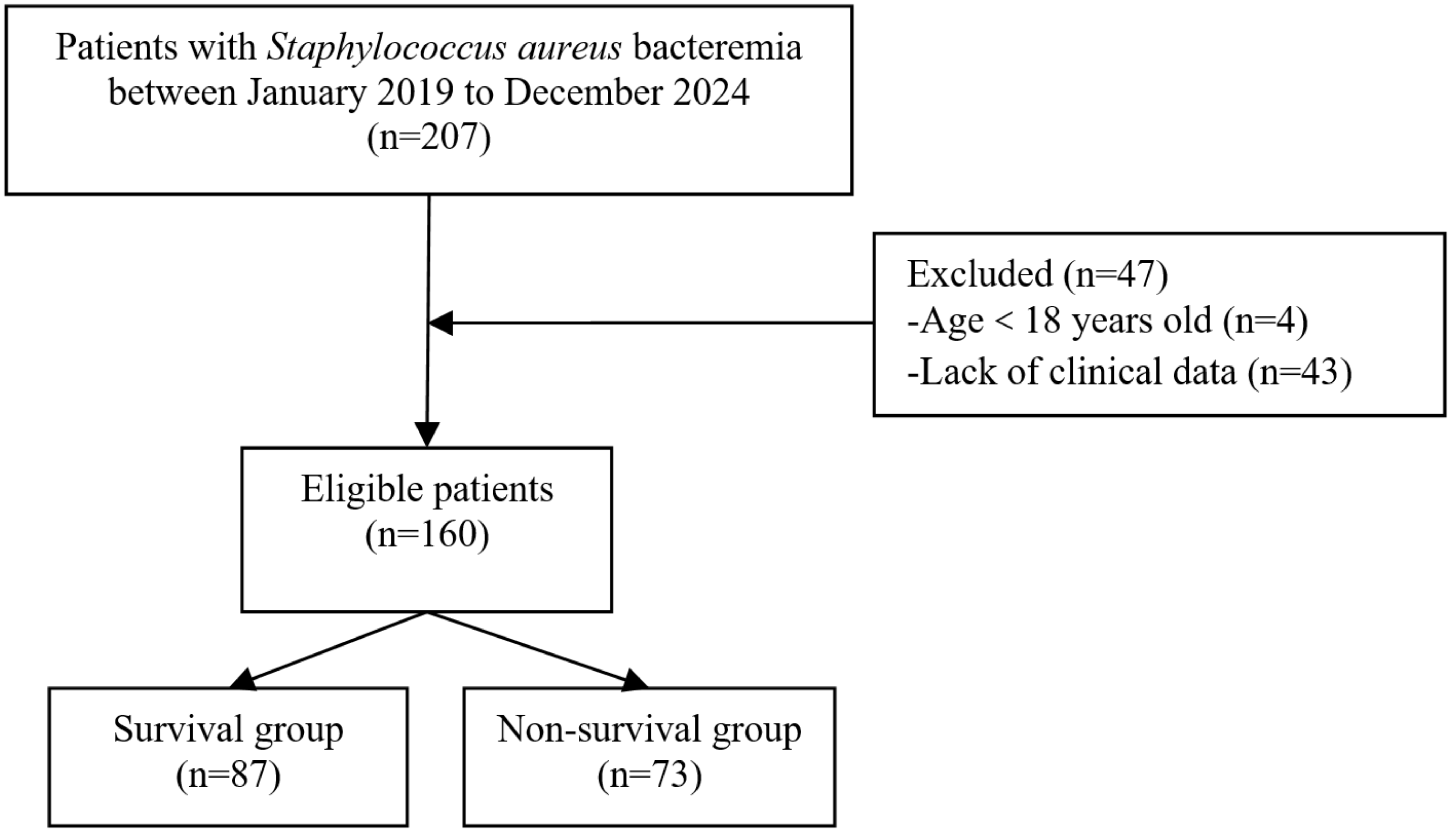
Patients flowchart.

This study was a single-center retrospective observational study. The study screened patients with SAB who were admitted to Beijing Luhe Hospital, Capital Medical University between January 2019 and December 2024. Inclusion criteria: (i) At least one set of blood cultures positive for *Staphylococcus aureus*; (ii) Clinically diagnosed with SAB (Inagaki et al., 2019); (iii) Age≥18 years. Exclusion criteria: (i) Age<18 years old; (ii) Lack of complete clinical data. A total of 207 patients with *Staphylococcus aureus* bloodstream infection were initially screened. Among them, 4 patients were excluded due to age<18 years, and 43 were excluded due to missing data on PaO_2_/FiO_2_ ratio and lactate. After the process, 160 patients were enrolled in the final analysis. Based on the in-hospital outcome, patients were categorized into the survival group (n=87) and the non-survival group (n=73) (**Figure 1**). The aim of this study was to identify independent risk factors for in-hospital mortality by comparing the clinical characteristics of the two groups. Subsequently, we developed and validated a predictive model for in-hospital mortality and evaluating its performance against a baseline model using the SOFA score alone. The study was approved by the ethics committee of Beijing Luhe Hospital (2025-LHKY-017-01). All patient data were collected from the hospital information system (HIS).

### 2.2 Clinical data

This study collected data on demographic characteristics, underlying comorbidities, clinical features, laboratory parameters, and clinical outcomes, with all data collection anchored to the time of SAB onset, which was defined as the date on which the first set of blood cultures was collected and subsequently yielded a positive result for *Staphylococcus aureus*. According to the bacteremia source, SAB episodes were classified into three categories: (1) Secondary bacteremia: presence of a definite local infection focus (e.g., pneumonia, urinary tract infection, intra-abdominal infection, skin and soft tissue infection), regardless of central venous catheter (CVC) placement; (2) Catheter-related bacteremia: presence of a CVC without any of the above definite infection foci; (3) Primary bacteremia: absence of both CVC and any definite infection focus. According to the acquisition setting, SAB episodes were classified into three categories: (1) Community-acquired SAB: positive blood culture obtained in the outpatient setting or within 48 hours of hospital admission, without recent hospitalization, indwelling catheter, or invasive procedure; (2) Healthcare-associated SAB: meeting the timing criteria for community-onset but with a history of hospitalization within the preceding 90 days, long-term dialysis, chemotherapy, or home infusion therapy; (3) Hospital-acquired SAB: positive blood culture obtained after 48 hours of hospital admission. The variables collected included: (1) age, sex; (2) underlying comorbidities: diabetes mellitus, ischemic heart disease, chronic pulmonary disease, cerebrovascular disease, heart failure, chronic liver disease, chronic kidney disease, immune-related disorders, active malignancy; (3) clinical information (all defined as status prior to blood culture collection): prolonged bedridden status (duration >1 month), history of broad-spectrum antibiotic use within 90 days, history of hospitalization within 90 days, pulmonary infection, urinary tract infection, intra-abdominal infection, skin and soft tissue infection, admission to the intensive care unit (ICU), central venous catheterization (CVC), invasive mechanical ventilation (MV), continuous renal replacement therapy (CRRT), and surgical procedure; (4) Laboratory parameters (all derived from the worst peripheral blood test results within a ±24-hour window around the time of the first positive blood culture collection). These included complete blood count: white blood cell count, neutrophil count, lymphocyte count, red blood cell distribution width (RDW), hemoglobin, platelet count; inflammatory markers: procalcitonin, C-reactive protein; biochemical parameters: albumin, serum creatinine, blood urea nitrogen, total bilirubin; coagulation function: D-dimer, fibrinogen, prothrombin time (PT), activated partial thromboplastin time (APTT); blood gas analysis: lactate, PaO_2_/FiO_2_ ratio; and microbiological feature: methicillin-resistant *Staphylococcus aureus* (MRSA) status; (5) Severity of illness score and clinical event: The Sequential Organ Failure Assessment (SOFA) score was calculated based on the same laboratory data source as item (4) (i.e., the worst values within the ±24-hour window around the time of the first positive blood culture collection). Shock was defined as the need for vasoactive agents to maintain blood pressure accompanied by hyperlactatemia, occurring within the same ±24-hour window; (6) clinical outcome: in-hospital mortality.

### 2.3 Statistical analysis

Continuous variables following a normal distribution were presented as mean ± standard deviation, and comparisons between groups were performed using the independent samples t-test. Non-normally distributed continuous variables were expressed as median (interquartile range), with group comparisons conducted using the Mann-Whitney U test. Categorical variables were presented as counts (percentages), and group differences were assessed using the chi-square test or Fisher’s exact test, as appropriate.

Univariate logistic regression analysis was employed to identify potential factors associated with in-hospital mortality. Variables with a *P* < 0.10 in the univariate analysis were included in the subsequent variable selection process. To mitigate the limitations of a single variable selection method, three approaches were applied: (1) best subset selection based on the Bayesian Information Criterion (BIC); (2) stepwise regression using the Akaike Information Criterion (AIC); and (3) LASSO regression with family=binomial, where the optimal tuning parameter λ was selected via five-fold cross-validation, applying the one-standard-error rule to determine lambda.1se. The variables jointly identified by all three methods were entered into multivariable logistic regression analysis, and those with *P* < 0.05 were retained in the final prediction model.

A multivariable logistic regression model was constructed using the selected predictors, and a nomogram was developed for visualization. Discrimination ability was assessed using the receiver operating characteristic (ROC) curve and the area under the curve (AUC). Calibration was evaluated using the Hosmer-Lemeshow goodness-of-fit test and a calibration plot. To account for optimism bias due to model overfitting, internal validation was performed using bootstrap resampling with 500 iterations on the final model. The optimism-corrected AUC was calculated, and a bias-corrected calibration curve was plotted.

The prediction model developed in this study (Model 2) was compared with a baseline model based on the Sequential Organ Failure Assessment (SOFA) score (Model 1). Discrimination ability of the two models was assessed using ROC and AUC. The difference in AUC between the two models was compared using bootstrap resampling with 500 iterations, with a two-sided *P* < 0.05 considered statistically significant. Calibration was evaluated using calibration plots and the Hosmer−Lemeshow goodness−of−fit test. Decision curve analysis (DCA) was employed to assess the clinical net benefit of the two models across a range of high-risk thresholds, and the results were compared with the default strategies of “treat all” and “treat none”. Statistical analyses were performed using Free Statistics analysis platform (Version 2.4, Beijing, China). Statistical significance was set at a two-sided *P* < 0.05 for final model coefficients and primary comparisons.

## 3 Results

### 3.1 Clinical characteristics

Initially, 207 patients with SAB were screened. Four patients were excluded due to age<18 years, and 43 were excluded due to missing data for key variables. Ultimately, 160 patients were enrolled in the primary analysis. Comparison between the included patients and the 43 excluded patients showed no significant difference in sex distribution (OR = 1.06, 95% CI: 0.52-2.16, *P* = 0.883), whereas significant differences were observed in age (OR = 0.98, 95% CI: 0.96-1.00, *P* = 0.045) and in-hospital mortality (2.3% vs. 45.6%; OR = 0.03, 95% CI: 0.00-0.21, *P* = 0.001). Among the 160 patients included, 66.2% (n=106) were male, with a mean age of 63.9 ± 15.3 years (range: 23–79 years). The all-cause in-hospital mortality rate was 45.6% (73/160), and the median length of hospital stay was 13.5 days (IQR: 7.0-32.2). MRSA accounted for 53.1% (85/160) of cases. The median hospital length of stay was significantly shorter in non-survivors than in survivors [8.0 (IQR: 3.0-15.0) vs. 22.0 (IQR: 12.0-48.5) days, *P* < 0.001]. According to the bacteremia source, secondary bacteremia was identified in 105 cases (65.6%), including 60 cases with an infection focus alone and 45 cases with both an infection focus and a central venous catheter; catheter-related bacteremia was identified in 55 cases (34.4%); and no cases of primary bacteremia were observed. In terms of acquisition setting, community-acquired SAB was identified in 14 cases (8.8%), healthcare-associated SAB in 63 cases (39.4%), and hospital-acquired SAB in 83 cases (51.9%). The corresponding in-hospital mortality rates were 7.1% (1/14), 46.0% (29/63), and 51.8% (43/83), respectively.

### 3.2 Factors associated with mortality in univariate analysis

Univariate logistic regression analysis identified multiple clinical and laboratory factors significantly associated with in-hospital mortality (**Table 1**). Regarding clinical characteristics, chronic liver disease, antibiotic use within the previous 90 days, intensive care unit admission, invasive mechanical ventilation, renal replacement therapy, and shock were associated with increased mortality risk (all *P* < 0.05). In terms of microbiological features, infection with MRSA strains was significantly associated with an increased risk of mortality (OR = 2.89, 95% CI: 1.51-5.53, *P* = 0.001). With respect to laboratory parameters, elevated red blood cell distribution width, decreased hemoglobin, decreased platelet count, elevated procalcitonin, decreased albumin, elevated urea, elevated total bilirubin, elevated D-dimer, prolonged prothrombin time, prolonged activated partial thromboplastin time, elevated lactate, decreased PaO_2_/FiO_2_ ratio, and higher SOFA score were significantly associated with increased mortality risk (all *P* < 0.05) (**Table 1**).

**Table 1:** Baseline clinical characteristics of patients with *Staphylococcus aureus* bacteremia.

Variables	Total (n=160)	Survivors (n=87)	Non-survivors (n=73)	*P*
Male, n (%)	106 (66.2)	59 (67.8)	47 (64.4)	0.648
Age, (years)	63.9 ± 15.3	61.8 ± 14.7	66.5 ± 15.8	0.054
Comorbidities				
Diabetes mellitus, n (%)	76 (47.5)	45 (51.7)	31 (42.5)	0.243
Ischemic heart disease, n (%)	48 (30.0)	22 (25.3)	26 (35.6)	0.157
Chronic pulmonary disease, n (%)	9 (5.6)	5 (5.7)	4 (5.5)	0.942
Cerebrovascular disease, n (%)	45 (28.1)	21 (24.1)	24 (32.9)	0.222
Heart failure, n (%)	42 (26.2)	19 (21.8)	23 (31.5)	0.168
Chronic liver disease, n (%)	14 (8.8)	3 (3.4)	11 (15.1)	0.017
Chronic kidney disease, n (%)	45 (28.1)	24 (27.6)	21 (28.8)	0.869
Hematologic disease, n (%)	2 (1.2)	0 (0.0)	2 (2.7)	0.988
Immunologic disease, n (%)	7 (4.4)	3 (3.4)	4 (5.5)	0.535
Malignant cancer, n (%)	22 (13.8)	10 (11.5)	12 (16.4)	0.368
Clinical information				
Long term bedridden, n (%)	17 (10.6)	9 (10.3)	8 (11.0)	0.900
Application of antibiotics <90 days, n (%)	78 (48.8)	33 (37.9)	45 (61.6)	0.003
History of hospitalization <90 days, n (%)	50 (31.2)	28 (32.2)	22 (30.1)	0.781
Pneumonia, n (%)	57 (35.6)	28 (32.2)	29 (39.7)	0.322
Urinary tract infection, n (%)	1 (0.6)	0 (0.0)	1 (1.4)	0.987
Intra-abdominal infection, n (%)	12 (7.5)	5 (5.7)	7 (9.6)	0.363
Skin and soft-tissue infection, n (%)	42 (26.2)	28 (32.2)	14 (19.2)	0.065
Intensive care unit admission, n (%)	106 (66.2)	42 (48.3)	64 (87.7)	<0.001
Placement of central venous catheters, n (%)	85 (53.1)	41 (47.1)	44 (60.3)	0.098
Invasive mechanical ventilation, n (%)	88 (55.0)	30 (34.5)	58 (79.5)	<0.001
Renal replacement therapy, n (%)	53 (33.1)	20 (23.0)	33 (45.2)	0.003
Surgical procedure, n (%)	45 (28.1)	27 (31.0)	18 (24.7)	0.372
Laboratory test at admission				
White blood cell count, (×10^9^/L)	12.8 ± 7.8	12.1 ± 6.7	13.6 ± 9.0	0.239
Neutrophil count, (×10^9^/L)	11.4 ± 7.6	10.7 ± 6.6	12.3 ± 8.5	0.197
Lymphocyte count, (×10^9^/L)	0.6 (0.4, 0.9)	0.6 (0.4, 0.9)	0.6 (0.4, 0.9)	0.897
RDW, (%)	15.4 ± 3.2	14.5 ± 2.2	16.5 ± 3.9	<0.001
Hemoglobin, (g/L)	95.9 ± 27.6	101.3 ± 27.0	89.4 ± 27.0	0.007
Platelet, (×10^9^/L)	171.8 ± 102.3	206.0 ± 104.2	131.0 ± 83.9	<0.001
C-reactive protein, (mg/L)	159.2 ± 87.2	149.7 ± 86.1	170.5 ± 87.9	0.134
Procalcitonin, (ng/mL)	5.5 (1.1, 15.6)	2.4 (0.8, 9.1)	10.3 (3.9, 20.5)	0.023
Albumin, (g/L)	28.8 ± 5.0	30.8 ± 4.7	26.3 ± 4.1	<0.001
Creatinine, (μmol/L)	122.0 (69.8, 320.5)	84.0 (59.5, 199.5)	187.0 (97.0, 334.0)	0.385
Urea, (mmol/L)	12.4 (6.5, 22.2)	9.0 (5.4, 13.9)	18.1 (10.8, 25.2)	<0.001
Total bilirubin, (μmol/L)	11.1 (6.9, 21.9)	9.1 (6.0, 15.3)	18.8 (8.5, 31.4)	0.008
D-Dimer, (μg/mL)	1.7 (0.8, 3.1)	1.1 (0.6, 2.2)	2.7 (1.3, 4.7)	<0.001
Fibrinogen, (g/L)	4.5 ± 1.9	4.7 ± 1.8	4.2 ± 2.0	0.146
Prothrombin time, (s)	15.8 ± 8.0	13.9 ± 4.3	18.0 ± 10.5	0.002
APTT, (s)	33.5 (29.9, 39.2)	32.2 (29.0, 36.5)	37.0 (31.0, 46.9)	0.015
Lactate, (mmol/L)	2.2 (1.5, 3.5)	1.8 (1.2, 2.3)	2.7 (2.1, 4.2)	0.001
PaO_2_/FiO_2_ ratio, (mmHg)	259.6 ± 99.6	313.5 ± 82.0	195.3 ± 78.5	<0.001
MRSA, n (%)	85 (53.1)	36 (41.4)	49 (67.1)	0.001
Shock, n (%)	88 (55.0)	23 (26.4)	65 (89.0)	<0.001
SOFA score	7.4 ± 4.9	3.8 ± 2.4	11.7 ± 3.3	<0.001

Data were presented as n (%) or mean ± SD, SD standard deviation. *P*-values represent comparisons between non-survivors and survivors. APTT, activated partial thromboplastin time; RDW, red blood cell distribution width; MRSA, methicillin-resistant *Staphylococcus aureus*.

### 3.3 Development and performance of the multivariable model

Univariate logistic regression analysis was employed to identify potential factors associated with in-hospital mortality. Variables with a *P* < 0.10 in the univariate analysis were included in the variable selection process.

#### 3.3.1 Variable selection results

**Figure 2:**
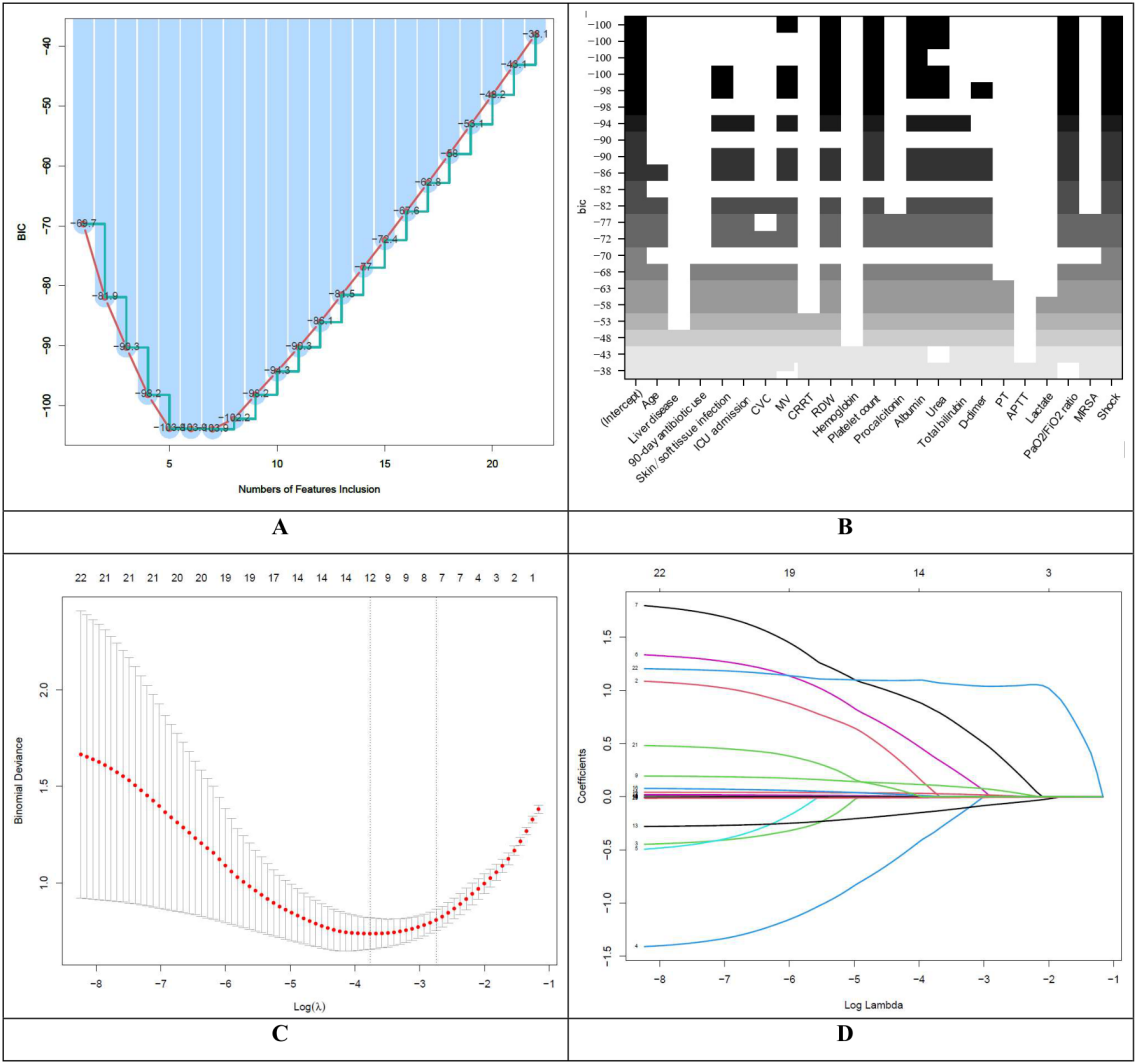
Variable selection. **(A, B)** Variable selection based on minimum Bayesian Information Criterion (BIC) **(C, D)** Variable selection based on Least Absolute Shrinkage and Selection Operator (LASSO) regression.

Best subset selection was employed to further screen variables with *P* < 0.10 in the univariate analysis. The optimal combination of predictive variables was determined by minimizing the Bayesian Information Criterion (BIC). Based on the minimum BIC value, the following variables were selected: Invasive mechanical ventilation, RDW, platelet count, albumin, urea, PaO_2_/FiO_2_ ratio, and shock (**Figures 2A, B**).

Stepwise regression based on the Akaike Information Criterion (AIC) was employed for variable selection. The stepwise procedure combined forward selection and backward elimination, with the goal of minimizing the AIC value. The following variables were ultimately included: CVC, Invasive mechanical ventilation, RDW, platelet count, albumin, urea, PaO_2_/FiO_2_ ratio, and shock. The AIC value of the final model was 104.409.

LASSO regression was employed for variable selection. The model was fitted with family=binomial, and the optimal tuning parameter λ was selected via 5−fold cross−validation based on the minimization of binomial deviance. According to the one−standard−error rule, λ.1se = 0.064 was chosen as the optimal parameter. At this λ value, seven variables with non−zero coefficients were retained, including invasive mechanical ventilation (coefficient = 0.376), RDW (coefficient = 0.058), platelet count (coefficient = -0.003), albumin (coefficient = -0.068), urea (coefficient = 0.012), PaO_2_/FiO_2_ ratio (coefficient = -0.006), and shock (coefficient = 1.04). Among these, invasive mechanical ventilation, RDW, urea, and shock were risk factors (positive coefficients), whereas platelet count, albumin, and PaO_2_/FiO_2_ ratio were protective factors (negative coefficients) (**Figures 2C, D**).

#### 3.3.2 Prediction model and internal validation

Variables jointly identified by all three methods were entered into multivariable logistic regression analysis. Multivariate logistic regression analysis showed that PaO_2_/FiO_2_ ratio (OR = 0.988, 95%CI: 0.980-0.996, *P* = 0.002), albumin (OR = 0.825, 95%CI: 0.724-0.941, *P* = 0.004), and platelet count (OR = 0.991, 95%CI: 0.985-0.997, *P* = 0.004) were protective factors; while urea (OR = 1.065, 95%CI: 1.007-1.127, *P* = 0.028), RDW (OR = 1.252, 95%CI: 1.023-1.532, *P* = 0.028), and invasive mechanical ventilation (OR = 4.738, 95%CI: 1.347-16.668, *P* = 0.015) were independent risk factors. The variable “shock” did not reach statistical significance (OR = 3.025, 95%CI: 0.820-11.159, *P* = 0.096) and was therefore excluded from the final prediction model (**Table 2**). The final model comprised six predictors: PaO_2_/FiO_2_ ratio, urea, albumin, RDW, invasive mechanical sventilation, and platelet count. Based on 73 in-hospital deaths, the events per variable (EPV) ratio was 12.2:1.

**Table 2:** Multivariable logistic regression model for predicting in-hospital mortality.

Variable	Adjusted OR (95% CI)	*P* value
Invasive mechanical ventilation (Yes vs. No)	4.738 (1.347, 16.668)	0.015
Shock (Yes vs. No)	3.025 (0.820, 11.159)	0.096
Platelet count, (×10^9^/L)	0.991 (0.985, 0.997)	0.004
RDW, (%)	1.252 (1.023, 1.532)	0.028
Albumin, (g/L)	0.825 (0.724, 0.941)	0.004
Urea, (mmol/L)	1.065 (1.007, 1.127)	0.028
PaO_2_/FiO_2_ ratio, (mmHg)	0.988 (0.980, 0.996)	0.002

OR, odds ratio; CI, confidence interval.

**Figure 3:**
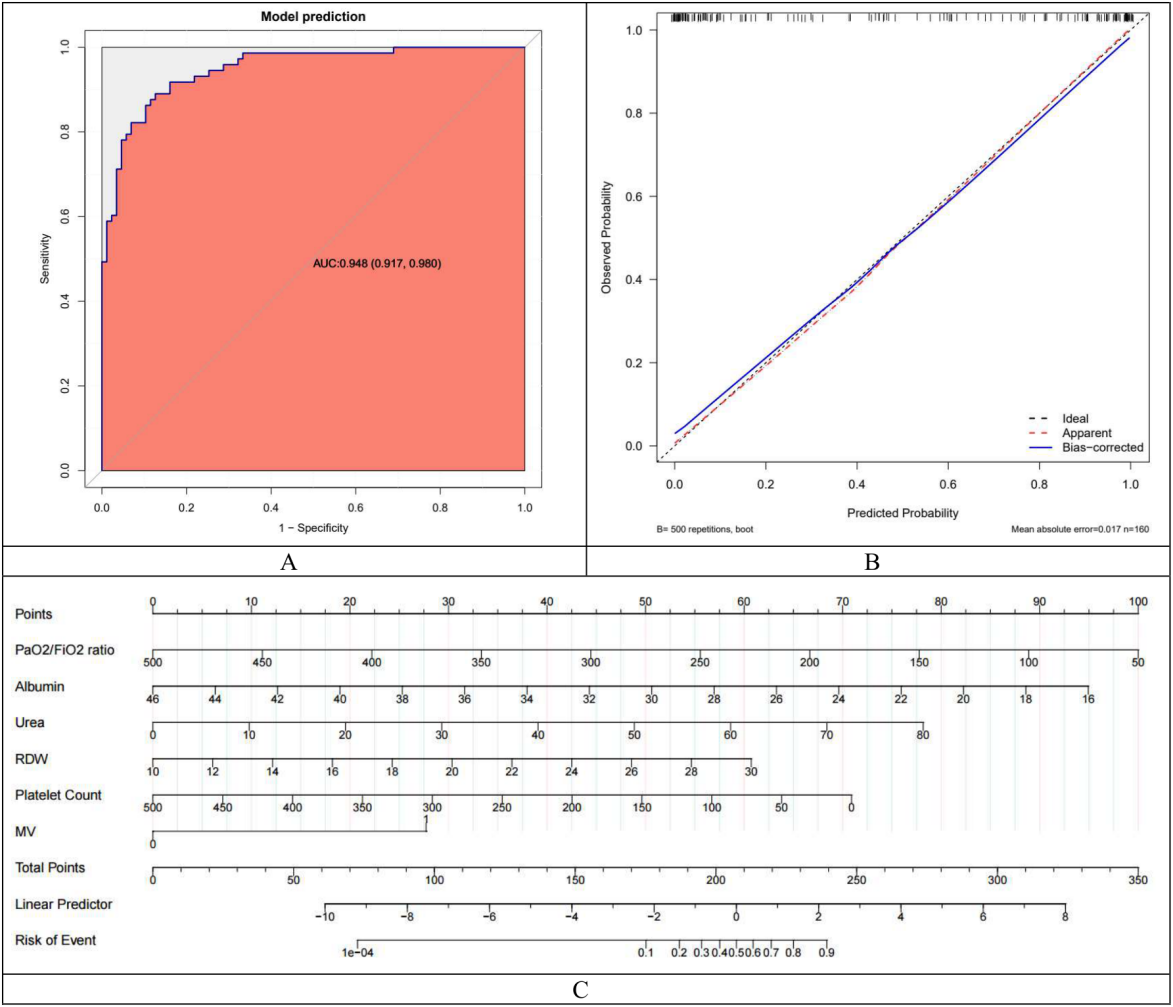
**(A)** Receiver operating characteristic (ROC) curve of the combined clinical predictor model. **(B)** Calibration curve of the model. The apparent curve and the bias-corrected curve both aligned closely with the ideal 45-degree diagonal line, indicating good model calibration. **(C)** Nomogram for predicting mortality risk based on the predictor model. The nomogram is used as follows: for each of the 6 variables, locate the patient’s value on the corresponding axis and draw a vertical line upward to the “Points” scale to determine the points assigned to that variable. Add the points from all 6 variables to obtain the total points. Finally, draw a vertical line downward from the “Total points” axis to the “Event risk” axis to obtain the predicted probability of in-hospital mortality.

The discriminative ability of the model was assessed using the ROC curve, which yielded an AUC of 0.948 (95% CI: 0.917-0.980) (**Figure 3A**). Bootstrap internal validation (500 resamples) further confirmed excellent discrimination, with a bias-corrected C-index of 0.934. Regarding calibration, the Hosmer-Lemeshow test yielded a *P* value of 0.658 (**Figure 3B**), with an R² of 0.735 and a Brier score of 0.090, indicating good calibration.

A nomogram incorporating six predictors (PaO_2_/FiO_2_ ratio, urea, albumin, RDW, MV, and platelet count) was constructed to provide individualized risk prediction (**Figure 3C**). The nomogram was used as follows: first, locate the patient’s specific value on each variable axis and draw a vertical line upward to the “Points” scale to obtain the points for each variable; second, sum the points from all six variables to obtain the total points; finally, draw a vertical line downward from the “Total points” axis to the “Event risk” axis to read the predicted probability of in-hospital mortality for that patient. Using a hypothetical patient with SAB as an example, the clinical parameters were as follows: PaO_2_/FiO_2_ ratio 150 mmHg, serum albumin 30 g/L, urea 20 mmol/L, RDW 14%, platelet count 150×10^9^/L, and the patient was receiving invasive mechanical ventilation. As shown in **Figure 3C**, the corresponding points for each variable were approximately 77.5 (PaO_2_/FiO_2_ ratio), 50 (albumin), 20 (urea), 12.5 (RDW), 50 (platelet count), and 27.5 (invasive mechanical ventilation), respectively, yielding a total point of approximately 237.5, which corresponded to a predicted risk of in-hospital mortality of approximately 87%.

### 3.4 Comparison with the SOFA score-based baseline model

**Figure 4:**
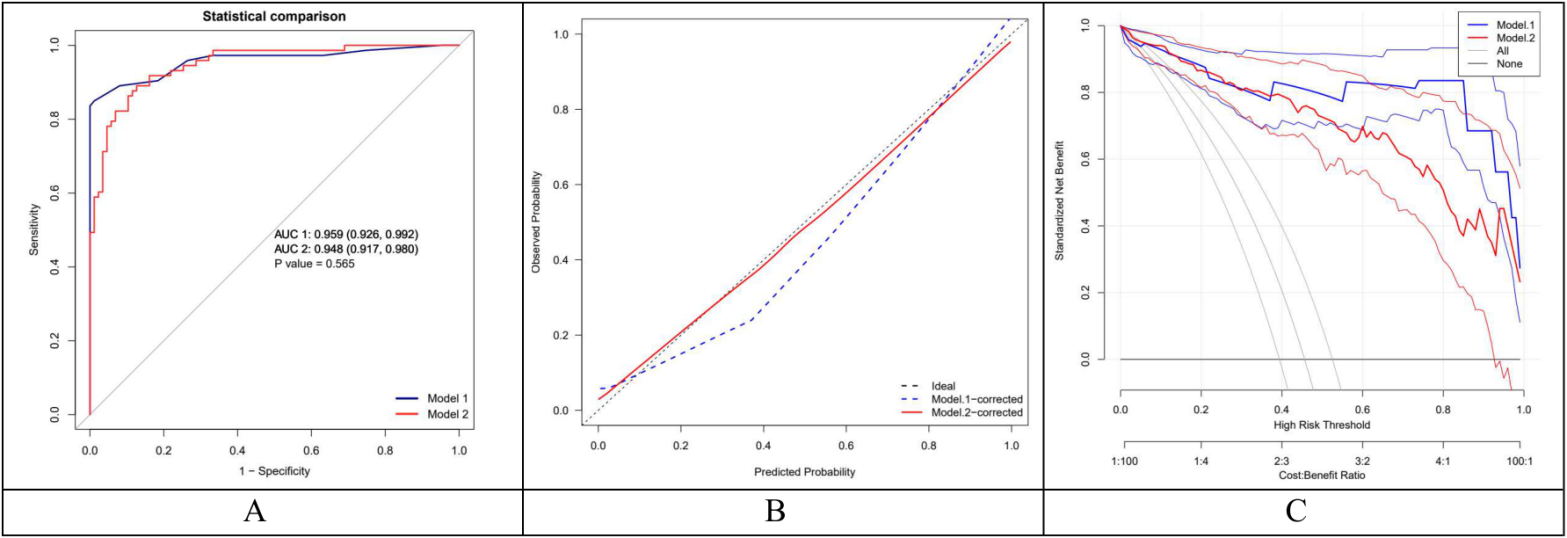
**(A)** Receiver operating characteristic (ROC) curves of Model 1 (SOFA score-based baseline model) and Model 2 (combined clinical predictor model). **(B)** Calibration curves of Model 1 and Model 2. The bias-corrected calibration curves for both models closely followed the ideal 45-degree diagonal line. **(C)** Decision curve analysis comparing the net benefit of Model 1 and Model 2 across a range of high-risk thresholds.

The performance of the prediction model (Model 2) was further evaluated by comparing with a baseline model (Model 1) based on the SOFA score. Discrimination was assessed using the area under the receiver operating characteristic curve (AUC). The detailed performance metrics are presented in **Table 3**. Model 1 achieved an AUC of 0.959 (95% CI: 0.926-0.992), with an optimal cut-off value of 0.647. At this threshold, the model yielded a sensitivity of 84.93%, specificity of 98.85%, and a Youden index of 0.838. Model 2 demonstrated comparable discriminatory ability, with an AUC of 0.948 (95% CI: 0.917-0.980) and an optimal cut-off value of 0.456. The corresponding sensitivity, specificity, and Youden index were 89.04%, 87.36%, and 0.764, respectively. No statistically significant difference was observed between the AUCs of the two models (bootstrap test with 500 resamples, *P* = 0.565), indicating that the combined clinical predictor model achieved similar predictive performance to the SOFA score-based baseline model (**Figure 4A**).

**Table 3:** Performance comparison between the baseline model and the combined clinical predictor model.

Model	AUC (95% CI)	Optimal cut-off	Sensitivity, %	Specificity, %	PPV, %	NPV, %	Youden index
Model 1	0.959(0.926-0.992)	0.647	84.93	98.85	98.41	88.66	0.838
Model 2	0.948 (0.917-0.980)	0.456	89.04	87.36	85.53	90.48	0.764
*P*-value	0.565						

AUC, area under the receiver operating characteristic curve; CI, confidence interval; PPV, positive predictive value; NPV, negative predictive value. The *P*−value compares the AUCs of the two models (bootstrap test, n=500).

Calibration was assessed using calibration plots and the Hosmer−Lemeshow goodness−of−fit test. As shown in **Figure 4B**, the corrected calibration curves for both models closely followed the ideal reference line. The Hosmer−Lemeshow test yielded a *P* value of 0.026 for Model 1 and 0.658 for Model 2. Model 2 demonstrated good agreement between predicted probabilities and observed outcomes, reflecting excellent calibration performance, whereas Model 1 showed a modest lack of fit despite acceptable graphical calibration.

Decision curve analysis revealed that Model 2 achieved net benefit comparable to Model 1 across the majority of high-risk thresholds (**Figure 4C**). Although Model 1 demonstrated a higher peak net benefit at lower thresholds, Model 2 maintained more stable clinical utility across an expanded threshold range, particularly at higher risk levels where Model 1’s net benefit declined more rapidly. Both models consistently outperformed the default “treat-all” and “treat-none” strategies.

## 4 Discussion

In our study, we integrated routine clinical variables to construct and validate a nomogram for predicting in-hospital mortality among patients with SAB. We systematically compared its performance against a baseline model derived from the SOFA score. The final model incorporated six independent predictors. Among these, the PaO_2_/FiO_2_ ratio, albumin, and platelet count were protective factors, while urea, RDW, and invasive mechanical ventilation were independent risk factors. The model exhibited excellent discrimination (AUC = 0.948) and calibration (Hosmer-Lemeshow test *P* = 0.658). Bootstrap internal validation yielded a bias-corrected C-index of 0.934, and the calibration curve closely aligned with the ideal diagonal line. The nomogram can be applied in routine clinical practice to assist clinicians in identifying high-risk patients with SAB at an early stage. By inputting the six readily available predictors, clinicians can obtain an individualized estimate of in-hospitalmortality risk, thereby facilitating timely risk stratification.

SAB is one of the bloodstream infections with the highest mortality rate worldwide. According to a large-scale systematic review and meta-analysis by Bai et al. (2022), the one-month mortality rate of SAB patients after 2011 was 18.1%, and the risk of death increased by approximately 4% for every 10% increase in the proportion of MRSA. The overall in-hospital mortality rate in the present study was 45.6%, which is significantly higher than the pooled estimate reported in the aforementioned meta-analysis. This discrepancy likely reflects the greater proportion of critically ill patients in our cohort: 66.2% were admitted to the intensive care unit, 55.0% presented with shock, and 55.0% received invasive mechanical ventilation. Notably, MRSA accounted for 53.1% of cases. Univariate analysis revealed that MRSA infection was significantly associated with in-hospital mortality (OR = 2.89, 95% CI: 1.51-5.53, *P* = 0.001). However, MRSA was not selected into the final prediction model by any of the variable selection methods employed (including BIC best subset selection, AIC stepwise regression, and LASSO regression), a finding that warrants further discussion. We speculate that this phenomenon may reflect the improved prognosis of MRSA infection resulting from standardized treatment. In our center, routine therapeutic drug monitoring of vancomycin is performed for patients with confirmed MRSA bloodstream infection, with dose adjustments based on pharmacokinetic/pharmacodynamic targets, and source control measures (e.g., catheter removal, debridement of infectious foci) are actively implemented. These standardized diagnostic and therapeutic strategies may have partially offset the excess mortality risk associated with MRSA, rendering the resistance phenotype alone no longer an independent predictor after multivariable adjustment. Furthermore, Özdemir et al. (2025) also found that MRSA infection had no independent impact on mortality in patients with SAB, suggesting that differences in the virulence of circulating MRSA strains, host immune status, and clinical management practices across regions may all influence the prognostic value of the resistance phenotype. As emphasized by Liu et al. in the IDSA guidelines (Liu et al., 2011), the management of MRSA infection requires a multidimensional approach encompassing host factors, source control, and antibiotic selection, all of which may be prognostic determinants beyond the resistance phenotype alone. Invasive mechanical ventilation was the strongest independent risk factor in the present study (OR = 4.738, 95% CI: 1.347-16.668). The study by Özdemir et al. (2025) identified MV as an important predictor of mortality in patients with SAB, which is highly consistent with our findings. Furthermore, each 1 mmHg increase in the PaO_2_/FiO_2_ ratio was associated with an approximately 1.2% reduction in mortality risk (OR = 0.988, 95% CI: 0.980-0.996, *P* = 0.002). This finding underscores the importance of early respiratory function protection and optimization of oxygenation status in the management of SAB. RDW is a commonly used indicator reflecting the heterogeneity of red blood cell size and has recently been increasingly recognized as an important biomarker of inflammation and oxidative stress (Slaughter et al., 2013; Xu et al., 2025). In the present study, each 1% increase in RDW was associated with a 25.2% increase in mortality risk among patients with SAB (OR = 1.252, 95% CI: 1.023-1.532, *P* = 0.028), suggesting that elevated RDW is closely associated with poor prognosis in this population. In the study by Yoon et al., compared with blood culture-negative sepsis, RDW was statistically significant in the blood culture-positive sepsis group, indicating that RDW may be more closely related to the systemic inflammatory response triggered by documented pathogen infection (Yoon et al., 2023). Omer and Mohammed reported that 92% of neonates with blood culture-proven sepsis had elevated RDW, which was significantly correlated with a positive CRP, further supporting RDW as an adjunctive indicator of infectious inflammation (Omer and Mohammed, 2021). Elevated urea (OR = 1.065, 95% CI: 1.007-1.127, *P* = 0.028) and decreased platelet count (OR = 0.991, 95% CI: 0.985-0.997, *P* = 0.004) are common manifestations of sepsis-related multiorgan dysfunction. Furthermore, each 1 g/L increase in albumin was associated with a 17.5% reduction in mortality risk (OR = 0.825, 95% CI: 0.724-0.941, *P* = 0.004). This finding is consistent with the study by Borcak et al. (2025), which included 356 patients with SAB and identified hypoalbuminemia as an independent risk factor for both 30-day and in-hospital mortality (HR = 11.76, *P* = 0.01). Özdemir et al. (2026) also reported that hypoalbuminemia was an independent predictor of in-hospital mortality in a study of 319 patients with septic shock (OR = 0.89, 95% CI: 0.84-0.94). Collectively, these findings underscore the central role of nutritional status and organ function reserve in the prognosis of SAB.

Our predictive model achieved an AUC of 0.948, and its calibration curve closely aligned with the ideal diagonal, indicating excellent discrimination and calibration. Xie et al. (2025) developed a nomogram to predict in-hospital mortality in SAB patients, with a mean AUC of 0.930. Our model demonstrated comparable predictive performance to theirs. When compared with the baseline model based on the SOFA score (Model 1), Model 1 yielded an AUC of 0.959, which was slightly higher than that of our model (0.948); however, the difference was not statistically significant (*P* = 0.565), suggesting that the two models have comparable overall discriminative ability. However, it is worth noting that the calibration of the SOFA model in this study was suboptimal (Hosmer-Lemeshow test, *P* = 0.026), indicating a systematic deviation between its predicted probabilities and the actual observed outcomes. This suggests that the SOFA score may have certain limitations in providing quantitative risk predictions at the individual level, further highlighting the need to develop a predictive model with improved calibration performance specifically for this population. Decision curve analysis showed that the net benefits of the two models were comparable across the majority of high-risk threshold ranges.

SAB exhibits significant clinical heterogeneity, and the “personalized medicine” paradigm constitutes a key objective in this field (Mudrik-Zohar et al., 2026). By integrating clinical variables, this study has enriched the dimensions of prognostic prediction for SAB. The six variables incorporated into the model are all routinely collected clinical parameters that can be rapidly obtained without the need for additional calculations. Furthermore, the use of a nomogram enables clinicians to intuitively assess the individualized mortality risk for each patient.

When interpreting the findings of this study, several limitations should be considered. First, the retrospective, single-center design of this study inherently introduces a risk of selection bias, which is a primary concern when interpreting the applicability of our model. During initial screening, 43 patients were excluded due to missing data on key variables (PaO_2_/FiO_2_ ratio and lactate). The in-hospital mortality rate in this excluded group was significantly lower than that in the included analysis cohort (2.3% vs. 45.6%; OR = 0.03, 95% CI: 0.00-0.21, *P* = 0.001), suggesting that excluded patients had milder clinical illness. This difference clearly indicates that the missing data were not completely random and that the exclusion process may have systematically removed patients with less severe disease. Consequently, the practical applicability of our prediction model is primarily limited to patients with complete arterial blood gas data (i.e., those with moderate-to-severe disease). Extrapolation of our findings to patients with milder illness or to different clinical settings should therefore be undertaken with great caution. Second, although the sample size in this study met the basic requirements for model development, the relatively limited sample size still constrained the statistical power of the analysis. Therefore, our preliminary findings should be interpreted with caution and require validation in larger, multicenter cohorts. Third, because the data were derived from real-world clinical practice, there may be some variability in the timing of certain laboratory measurements, despite our use of a ±24hour window to minimize this effect. This variability could lead to data bias. Fourth, this study did not include treatment-related variables, such as source control (e.g., catheter removal, debridement of infectious foci) and antibiotic therapy (timing, duration, and appropriateness for MRSA). These variables are important prognostic factors for outcomes of Staphylococcus aureus bloodstream infection. However, due to the single-center retrospective design, the completeness and standardization of treatment information documented in medical records were insufficient, and the proportion of missing data was high, precluding a meaningful sensitivity analysis. Therefore, we were unable to incorporate these variables into model development. Future prospective studies with standardized collection of treatment-related variables are needed to more comprehensively assess their impact on prognosis. Fifth, we performed bootstrap internal validation with 500 resamples. The bias-corrected C-index was 0.934, and the calibration curve demonstrated satisfactory performance. Nevertheless, given the single-center retrospective design and relatively small sample size, a certain degree of overfitting cannot be entirely ruled out. Sixth, in the classification of bacteremia sources, due to the inherent limitations of the retrospective study design, we were only able to document the presence of clinical factors (e.g., pneumonia, catheter placement) but could not determine their homology with SAB. Future prospective studies with predefined diagnostic criteria and rigorous causal assessment are needed to validate the findings of this study. Seventh, the lack of external validation means that the generalizability and stability of our model in broader populations remain to be confirmed. Future multicenter, prospective cohort studies are needed to externally validate our model.

In this study, we constructed and validated a predictive model for in-hospital mortality risk in patients with SAB. The model demonstrated excellent discrimination and calibration, with predictive performance comparable to that of the SOFA score. This nomogram may serve as a practical clinical tool to facilitate early identification of SAB patients at high risk of mortality and to guide individualized treatment decisions.

## Data Availability

The original contributions presented in the study are included in the article/supplementary material. Further inquiries can be directed to the corresponding author.
